# Heat-Driven Synchronization in Coupled Liquid Crystal Elastomer Spring Self-Oscillators

**DOI:** 10.3390/polym15163349

**Published:** 2023-08-09

**Authors:** Kai Li, Haiyang Wu, Biao Zhang, Yuntong Dai, Yong Yu

**Affiliations:** Department of Civil Engineering, Anhui Jianzhu University, Hefei 230601, China

**Keywords:** synchronization, liquid crystal elastomer, spring oscillator, linear temperature field, fiber

## Abstract

Self-oscillating coupled machines are capable of absorbing energy from the external environment to maintain their own motion and have the advantages of autonomy and portability, which also contribute to the exploration of the field of synchronization and clustering. Based on a thermally responsive liquid crystal elastomer (LCE) spring self-oscillator in a linear temperature field, this paper constructs a coupling and synchronization model of two self-oscillators connected by springs. Based on the existing dynamic LCE model, this paper theoretically reveals the self-oscillation mechanism and synchronization mechanism of two self-oscillators. The results show that adjusting the initial conditions and system parameters causes the coupled system to exhibit two synchronization modes: in-phase mode and anti-phase mode. The work conducted by the driving force compensates for the damping dissipation of the system, thus maintaining self-oscillation. The phase diagrams of different system parameters are drawn to illuminate the self-oscillation and synchronization mechanism. For weak interaction, changing the initial conditions may obtain the modes of in-phase and anti-phase. Under conditions of strong interactions, the system consistently exhibits an in-phase mode. Furthermore, an investigation is conducted on the influence of system parameters, such as the LCE elastic coefficient and spring elastic coefficient, on the amplitudes and frequencies of the two synchronization modes. This study aims to enhance the understanding of self-oscillator synchronization and its potential applications in areas such as energy harvesting, power generation, detection, soft robotics, medical devices and micro/nanodevices.

## 1. Introduction

Self-oscillation refers to the phenomenon where a system generates sustained oscillations or periodic changes without external excitation, due to internal coupling and feedback mechanisms [1,2,3,4,5,6,7]. As a result, self-oscillating systems do not require a continuous energy supply from external sources, reducing energy consumption and system complexity. These systems can be adjusted and controlled by tuning internal parameters and coupling methods. Additionally, self-oscillating systems exhibit great flexibility capable of displaying various oscillatory behaviors such as periodic oscillations [8,9] and chaotic oscillations [10,11,12]. Various feedback mechanisms have been suggested to counteract energy loss attributed to damping dissipation, including the coupling of chemical reactions and large deformations [13,14,15], as well as the self-shading mechanism [16]. Currently, self-oscillation systems are widely used in various scientific and engineering fields, such as sensor technology [10,17,18,19,20,21,22], soft robots [23,24] and so on.

In recent years, the exploration of active materials has further expanded the possibilities of self-oscillating systems. Through ongoing research and development, scientists continue to discover new active materials with unique properties and enhanced performance, such as dielectric elastomers [25], hydrogels [26,27], ionic gels [13], thermal response polymers [28] and liquid crystal elastomer (LCE) [29,30,31]. These active substances produce different responses when stimulated by light [7], heat [9], electricity [32] and magnetism [33], and a variety of self-sustained motion modes have been established according to this property, for example, torsion [34,35], vibration [6,36,37], bending [38,39,40,41,42], swing [43], rolling [44], oscillating [45], jumping [46,47], rotation [48], buckling [49,50], twisting [51], stretching [52], eversion or inversion [53] and other movements. LCE, a unique material, possesses a hybrid nature that combines the characteristics of both liquid crystals and elastomers, which constitutes a polymer network structure resulting from the cross-linking of liquid crystal monomer molecules. It is capable of making reversible morphological changes when exposed to external stimuli, including light, electricity, heat and magnetism. Therefore, LCE as a driver and deformation structure has the advantages of fast feedback speed, simple control, low noise [54,55,56,57,58] and so on. At present, a lot of work has been carried out on the experimental and theoretical research of self-oscillation based on LCE.

Despite the extensive research conducted on individual self-oscillator, there is still much to explore regarding the interplay and collective effect of multiple self-oscillators. Synchronization is found everywhere in nature, from the cardiac pacemaker cells to the migration of species, from pendulums to musical instruments, power systems, and lasers, and has many practical applications in electrical and mechanical engineering [59,60,61,62,63]. The roots of synchronization research date back to the 17th-century observation and discovery by the Dutch scientist Huygens of the synchronous behavior of two coupled pendulums [64]. He found that when two coupled pendulums are located on the same beam, they oscillate synchronously in opposite directions. Finally, it is found that the anti-phase synchronization between two pendulums is achieved by the tiny vibrations propagated by the beam, that is, by beam coupling [65]. In recent years, there are more and more research projects on how to apply the synchronization phenomenon to various fields, such as nonlinear systems [66,67,68] and complex network synchronization behavior [69,70,71,72].

Currently, there are few research studies on the interaction and synchronization of self-oscillation systems with multiple responsive materials. Li et al. investigated the synchronization of two coupled light-driven self-oscillating LCE pendulums [7]. The collective motion of two joint LCE oscillators based on the self-shadowing effect was investigated by Du et al. [73]. To achieve more functions and applications, further investigation is warranted to explore the synchronization phenomenon and its underlying mechanisms. This paper is based on the study of thermally responsive LCE spring self-oscillators in a linear temperature field, focusing on the synchronization behavior of two identical self-oscillators connected by springs and their mechanism. Based on the well-established dynamic LCE model [74,75,76], the mechanisms of self-oscillation and synchronization are theoretically investigated. The revelation of the interaction and synchronization mechanism among multiple self-oscillators is helpful to enhance the understanding of self-oscillator synchronization and its potential applications in areas such as energy harvesting, power generation, detection, soft robotics, medical devices and micro/nanodevices. In addition, the research in this paper has the potential to be extended to large-scale synchronization systems containing a large number of coupled oscillators, which has a promising application in this field.

The structure of this document is as follows. In Section 2, the dynamic model of the LCE spring oscillator is developed in conjunction with a well-established LCE dynamic model, and the governing equations of the synchronized system under a linear temperature field are derived. In Section 3, two synchronization modes of self-oscillation are proposed and the mechanisms of self-oscillation and synchronization are elaborated. In Section 4, the effects of the LCE elastic coefficient, spring elastic coefficient, thermal expansion coefficient, temperature gradient, first damping coefficient, second damping coefficient and characteristic time on the amplitude and frequency of the synchronization system are studied in detail. Finally, the conclusions of this paper are summarized in Section 5.

## 2. Model and Theoretical Formulation

In the current section, a coupled self-oscillating system consisting of two LCE fibers and a linear spring under a linear temperature field is proposed. Meanwhile, the governing equations and solution methods of the system are given.

### 2.1. Dynamic Model of Two LCE Spring Oscillators

**Figure 1:**
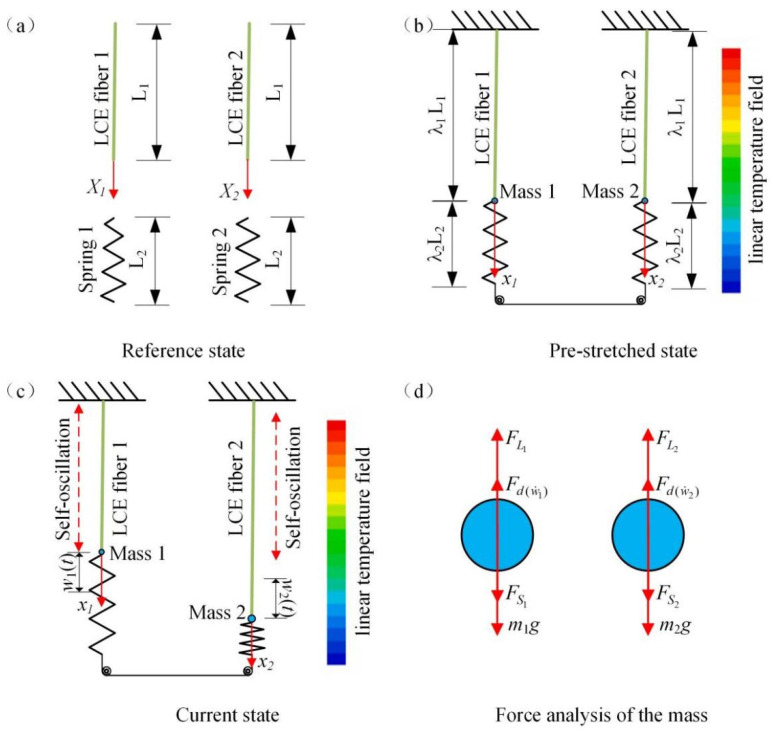
Schematic diagram of two identical LCE fibers connected by two identical springs within the linear temperature field. (**a**) Reference state; (**b**) pre-stretched state; (**c**) current state; (**d**) force analysis of the mass. Two coupled LCE oscillators can vibrate synchronously within the linear temperature field.

Figure 1 illustrates the coupled self-oscillating system within a linear temperature field, which consists of two identical LCE spring oscillators connected by two springs. In the non-stress state, the primary length of the LCE fiber is $L_{1}$ and the primary length of the spring is $L_{2}$, as shown in Figure 1a. According to Yakacki et al. [28], LC monomer (RM257) and cross-linking agent (PETMP), etc., are used as raw materials, and LCE fibers can be made by a two-step cross-linking reaction. First, one end of the LCE fiber is fixed, while another end is connected with a spring. The lower end of the spring is connected with another spring through a fixed pulley so that the two LCE fibers can be connected in series. To ensure that the system is force stabilized, the LCE fiber and the spring should be pre-stretched, where the prestretch amount is $\lambda_{1}$, $\lambda_{2}$, respectively. In the state of equilibrium, the lengths of the LCE fibers and the springs are $\lambda_{1}L_{1}$ and $\lambda_{2}L_{2}$, respectively, as shown in Figure 1b. Then, the equilibrium equation of the system in the non-stress state can be obtained:

(1) $$\left\{\begin{array}{l} m_{1}g+F_{s_{1}0}-F_{L_{1}0}=0\\ m_{2}g+F_{s_{2}0}-F_{L_{2}0}=0 \end{array}\right.,$$

where $F_{s}{}_{{}_{1}0}$ and $F_{s}{}_{{}_{2}0}$ are the initial elastic forces of the two springs, respectively; $F_{L}{}_{{}_{1}0}$ and $F_{L}{}_{{}_{2}0}$ are the initial elastic forces of two LCE fibers, respectively, where $F_{s0}=k(\lambda_{2}L_{2}-L_{2})$, $F_{L0}=K(\lambda_{1}L_{1}-L_{1})$. k and K are elastic coefficients of spring and LCE fiber, respectively.

In this case, we can obtain the relationship between *λ*_1_ and *λ*_2_, i.e.:

(2) $$\lambda_{2}=\frac{\bar{K}(\lambda_{1}-1)-1}{\bar{k}{\bar{L}}_{2}}\;+1,$$

where ${\bar{F}}_{s_{1}0}=F_{s_{1}0}/mg$, ${\bar{F}}_{s_{2}0}=F_{s_{2}0}/mg$, ${\bar{F}}_{L_{1}0}=F_{L_{1}0}/mg$, ${\bar{F}}_{L_{2}0}=F_{L_{2}0}/mg$, $\bar{k}=kL_{1}/mg$, $\bar{K}=KL_{1}/mg$ and ${\bar{L}}_{2}=L_{2}/L_{1}$.

When placed in the linear temperature field, LCE fibers begin to oscillate along the vertical direction, in which the displacements of particles 1 and 2 are $w_{1}(t)$ and $w_{2}(t)$, respectively, as shown in Figure 1c. The force analysis diagram of the two particles is given in Figure 1d, where $F_{s}{}_{{}_{1}}$ and $F_{s}{}_{{}_{2}}$ are the elastic force of the two springs, respectively (referred to as spring force); $F_{L}{}_{{}_{1}}$ and $F_{L}{}_{{}_{2}}$ are the elastic force of two fibers (hereinafter referred to as the driving force); and $F_{d}({\dot{w}}_{1})$ and $F_{d}({\dot{w}}_{2})$ are the damping force in the process of vibration. To simplify the analysis, we make the assumption that the damping force is directly proportional to the particle’s velocity and always acts in the opposite direction to the particle’s motion. The dynamic governing equations of the system can then be obtained and can be applied at any time:

(3) $$\left\{\begin{array}{l} m_{1}{\ddot{w}}_{1}(t)-m_{1}g-F_{s_{1}}(t)+F_{L_{1}}(t)+F_{d}({\dot{w}}_{1})=0\\ m_{2}{\ddot{w}}_{2}(t)-m_{2}g-F_{s_{2}}(t)+F_{L_{2}}(t)+F_{d}({\dot{w}}_{2})=0 \end{array}\right.,$$

where ${\ddot{w}}_{1}(t)$, ${\ddot{w}}_{2}(t)$ indicate the particle’s acceleration $\frac{d^{2}w_{1}(t)}{dt^{2}}$, $\frac{d^{2}w_{2}(t)}{dt^{2}}$, ${\dot{w}}_{1}(t)$, ${\dot{w}}_{2}(t)$ is the particle’s velocity $\frac{dw_{1}(t)}{dt}$, $\frac{dw_{2}(t)}{dt}$, and the spring force is $F_{s_{1}}=k[\lambda_{2}L_{2}-L_{2}-w_{1}(t)-w_{2}(t)]$, $F_{s_{2}}=k[\lambda_{2}L_{2}-L_{2}-w_{2}(t)-w_{1}(t)]$.

Since the system can vibrate continuously without divergence, only nonlinear damping is studied, and it is assumed that:

(4) $$\left\{\begin{array}{l} F_{d}({\dot{w}}_{1})=(a_{0}+a_{1}\left|{\dot{w}}_{1}\right|){\dot{w}}_{1}\\ F_{d}({\dot{w}}_{2})=(a_{0}+a_{1}\left|{\dot{w}}_{2}\right|){\dot{w}}_{2} \end{array}\right.,$$

where $a_{0}$ and $a_{1}$ represent the first and second damping coefficients, respectively.

### 2.2. Tension in the LCE Fibers

According to the non-uniform deformation of LCE fiber in a linear temperature field, the Lagrangian coordinate system $X_{1}$, $X_{2}$ and Euler coordinate system $x_{1}$, $x_{2}$ need to be established by taking the particle at the end of LCE fiber as the origin, as shown in Figure 1a,b. When LCE fiber vibrates, the instantaneous position and displacement of a particle can be used as $x_{i}=x_{i}(X_{i},t)(i=1,2)$, $u_{i}(X_{i},t)(i=1,2)$. The displacement of the particle is represented by $w_{1}(t)$ and $w_{2}(t)$, respectively.

We assume that the driving force of LCE fiber is linearly dependent on strain:

(5) $$\left\{\begin{array}{l} F_{L_{1}}=KL_{1}\left[\varepsilon_{1}\left(X,t\right)-\varepsilon_{T}\left(X,t\right)\right]\\ F_{L_{2}}=KL_{2}\left[\varepsilon_{2}\left(X,t\right)-\varepsilon_{T}\left(X,t\right)\right] \end{array}\right.,$$

where K is the elastic coefficient of LCE fiber, and one-dimensional strain $\varepsilon_{1}\left(X,t\right)$, $\varepsilon_{2}\left(X,t\right)$ is given by:

(6) $$\left\{\begin{array}{l} \varepsilon_{1}\left(X,t\right)=\frac{\partial u_{1}(X,t)}{\partial X_{1}}\\ \varepsilon_{2}\left(X,t\right)=\frac{\partial u_{2}(X,t)}{\partial X_{2}} \end{array}\right..$$

We assume that the heat-induced strain $\varepsilon_{T}(X,t)$ is linearly related to the temperature difference T(X,t) in LCE fiber:

(7) $$\varepsilon_{T}(X,t)=\alpha T(X,t),$$

where α represents the coefficient of thermal expansion, α<0 represents thermal contraction, and α>0 represents thermal expansion.

Since the driving force $F_{L}(t)$ is uniform and constant in the LCE fiber, it can be obtained by integrating both sides of Equation (5) from 0 to X and combining with Equations (6) and (7); the driving force at the end X=L of the LCE fiber can be written as:

(8) $$\left\{\begin{array}{l} F_{L_{1}}(t)=K\left[w_{1}(t)-\alpha \int_{0}^{L}T\left(X,t\right)dX\right]\\ F_{L_{2}}(t)=K\left[w_{2}(t)-\alpha \int_{0}^{L}T\left(X,t\right)dX\right] \end{array}\right..$$

Since the temperature field in LCE fiber is unevenly distributed and changes with time, heat exchange occurs between the fiber and its surroundings, resulting in a temperature distribution denoted by $T_{ext}(t)$. For simplicity, there is an assumption that the radius R is much smaller than the length L so that the temperature field in the LCE fiber can be seen as uniform, i.e., T=T(X,t). In this case, the temperature in the fiber can be obtained:

(9) $$\tau \frac{dT(X,t)}{dt}=T_{ext}(x)-T(X,t),$$

where $\tau =\frac{\rho_{c}}{h}$ indicates the characteristic time, $\rho_{c}$ is the heat capacity per unit length of the fiber, and h is the heat transfer coefficient. Assume that the steady-state temperature field in the environment is linear:

(10) $$T_{ext}(x)=\beta x+Q,$$

where Q refers to the temperature at x=0 and β represents the gradient of temperature.

By defining the following infinitesimal constants: $\bar{t}=t/\sqrt{L/g}$, ${\bar{F}}_{L}=F_{L}/mg$, $\bar{u}=u/L$, $\bar{w}=w/L$, $\bar{X}=X/L$, $\bar{x}=x/l$, $\bar{\tau }=\tau /\sqrt{L/g}$, $\bar{K}=KL/mg$, $\bar{\alpha }=\alpha T_{L}$, $\bar{T}=T/T_{L}$, ${\bar{T}}_{ext}=T_{ext}/T_{L}$, $\bar{\beta }=\beta L/T_{L}$ and $\bar{Q}=Q/T_{L}$ ($T_{L}$ is the temperature at x=L).

Thus, the elastic force of LCE fiber can be obtained

(11) $$\left\{\begin{array}{l} {\bar{F}}_{L_{1}}=\bar{K}\left[{\bar{w}}_{1}(\bar{t})-\bar{\alpha }\int_{0}^{1}\bar{T}({\bar{X}}_{1},\bar{t})d\bar{X}\right]\\ {\bar{F}}_{L_{2}}=\bar{K}\left[{\bar{w}}_{2}(\bar{t})-\bar{\alpha }\int_{0}^{1}\bar{T}({\bar{X}}_{2},\bar{t})d\bar{X}\right] \end{array}\right..$$

The solution of the temperature field is [77]:

(12) $$\bar{T}(\bar{X},\bar{t})=\frac{\bar{\beta }\left[\bar{w}(\bar{t})+1\right]}{e^{\bar{\alpha }\bar{\beta }}-1}\left(e^{\bar{\alpha }\bar{\beta }\bar{X}}-1\right)+\bar{Q}+\bar{\tau }\frac{\bar{\beta }\dot{w}(\bar{t})}{e^{\bar{\alpha }\bar{\beta }}-1}\left[\frac{\left(e^{\bar{\alpha }\bar{\beta }\bar{X}}-1\right)\left(\bar{\alpha }\bar{\beta }e^{\bar{\alpha }\bar{\beta }}-e^{\bar{\alpha }\bar{\beta }}+1\right)}{e^{\bar{\alpha }\bar{\beta }}-1}-\bar{\alpha }\bar{\beta }\bar{X}e^{\bar{\alpha }\bar{\beta }\bar{X}}\right].$$

By substituting Equation (12) into Equation (11), the elastic force ${\bar{F}}_{L}(t)$ of LCE fiber can be obtained:

(13) $$\left\{\begin{array}{l} {\bar{F}}_{L_{1}}(\bar{t})=\frac{\bar{K}\bar{\alpha }\bar{\beta }}{e^{\bar{\alpha }\bar{\beta }}-1}{\bar{w}}_{1}(\bar{t})+\bar{K}\bar{\alpha }\bar{\beta }\bar{\tau }\frac{1-e^{\bar{\alpha }\bar{\beta }}+\bar{\alpha }\bar{\beta }e^{\bar{\alpha }\bar{\beta }}}{{\left(e^{\bar{\alpha }\bar{\beta }}-1\right)}^{2}}{\dot{w}}_{1}(t)+\bar{K}\left(\frac{\bar{\alpha }\bar{\beta }}{e^{\bar{\alpha }\bar{\beta }}-1}-1-\bar{\alpha }\bar{Q}\right)\\ {\bar{F}}_{L_{2}}(\bar{t})=\frac{\bar{K}\bar{\alpha }\bar{\beta }}{e^{\bar{\alpha }\bar{\beta }}-1}{\bar{w}}_{2}(\bar{t})+\bar{K}\bar{\alpha }\bar{\beta }\bar{\tau }\frac{1-e^{\bar{\alpha }\bar{\beta }}+\bar{\alpha }\bar{\beta }e^{\bar{\alpha }\bar{\beta }}}{{\left(e^{\bar{\alpha }\bar{\beta }}-1\right)}^{2}}{\dot{w}}_{2}(t)+\bar{K}\left(\frac{\bar{\alpha }\bar{\beta }}{e^{\bar{\alpha }\bar{\beta }}-1}-1-\bar{\alpha }\bar{Q}\right) \end{array}\right..$$

### 2.3. Governing Equations

By defining ${\bar{F}}_{d}=F_{d}/mg$, ${\bar{a}}_{0}=\frac{a_{0}}{m}\sqrt{\frac{L}{g}}$, ${\bar{a}}_{1}=\frac{a_{1}L}{m}$, and combing with Equations (4) and (13), Equation (3) can be rewritten as:

(14) $$\left\{\begin{array}{l} {\ddot{w}}_{1}(\bar{t})-1-k(\lambda_{2}{\bar{L}}_{2}-{\bar{L}}_{2}-{\bar{w}}_{1}(\bar{t})-{\bar{w}}_{2}(\bar{t}))+\frac{\bar{K}\bar{\alpha }\bar{\beta }}{e^{\bar{\alpha }\bar{\beta }}-1}\left[{\bar{w}}_{1}(\bar{t})+\lambda_{1}-1\right]\\ \;+\bar{K}\bar{\alpha }\bar{\beta }\bar{\tau }\frac{1-e^{\bar{\alpha }\bar{\beta }}+\bar{\alpha }\bar{\beta }e^{\bar{\alpha }\bar{\beta }}}{{\left(e^{\bar{\alpha }\bar{\beta }}-1\right)}^{2}}{\dot{w}}_{1}(\bar{t})+\bar{K}\left(\frac{\bar{\alpha }\bar{\beta }}{e^{\bar{\alpha }\bar{\beta }}-1}-1-\bar{\alpha }\bar{Q}\right)+({\bar{a}}_{0}+{\bar{a}}_{1}\left|{\dot{w}}_{1}(\bar{t})\right|){\dot{w}}_{1}(\bar{t})=0\\ {\ddot{w}}_{2}(\bar{t})-1-k(\lambda_{2}{\bar{L}}_{2}-{\bar{L}}_{2}-{\bar{w}}_{2}(\bar{t})-{\bar{w}}_{1}(\bar{t}))+\frac{\bar{K}\bar{\alpha }\bar{\beta }}{e^{\bar{\alpha }\bar{\beta }}-1}\left[{\bar{w}}_{2}(\bar{t})+\lambda_{1}-1\right]\\ \;+\bar{K}\bar{\alpha }\bar{\beta }\bar{\tau }\frac{1-e^{\bar{\alpha }\bar{\beta }}+\bar{\alpha }\bar{\beta }e^{\bar{\alpha }\bar{\beta }}}{{\left(e^{\bar{\alpha }\bar{\beta }}-1\right)}^{2}}{\dot{w}}_{2}(\bar{t})+\bar{K}\left(\frac{\bar{\alpha }\bar{\beta }}{e^{\bar{\alpha }\bar{\beta }}-1}-1-\bar{\alpha }\bar{Q}\right)+({\bar{a}}_{0}+{\bar{a}}_{1}\left|{\dot{w}}_{2}(\bar{t})\right|){\dot{w}}_{2}(\bar{t})=0 \end{array}\right..$$

Equation (14) is an ordinary differential equation with second-order variable coefficients, which is difficult to obtain its analytic solution. In this case, the classical fourth-order Runge–Kutta method is adopted to solve Equation (14) numerically, and the steady-state response of LCE fiber is obtained, meaning, the time-history curve of oscillation of the system.

## 3. Two Modes of Synchronization and Their Mechanisms

In the current section, two synchronization modes, namely in-phase mode and anti-phase mode, are proposed according to the dynamic Equation (14), and the self-oscillation mechanism and synchronization mechanism are elaborated in detail.

To better study the synchronization behaviors of two LCE spring oscillators, it is necessary to obtain the typical values of the dimensionless system parameters. According to the existing experiments [52,54,78,79], the actual values of system parameters are summarized in Table 1, and the dimensionless system parameters are calculated in Table 2.

### 3.1. Two Synchronization Modes

**Figure 2:**
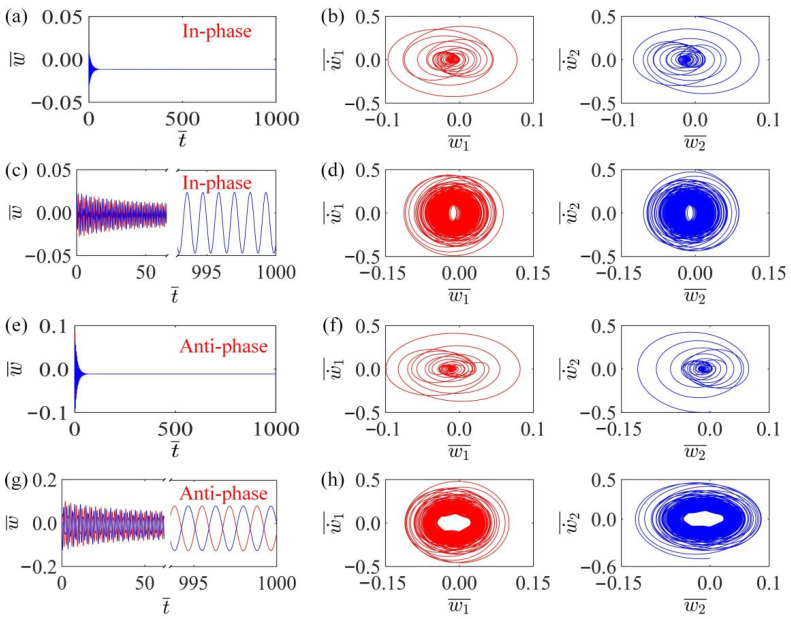
Two synchronization modes and two motion states of the thermal response LCE self-excited oscillator. The parameters are $\bar{K}=14$, $\bar{k}=8$, $\bar{\alpha }=-0.5$, $\bar{\beta }=0.1$, ${\bar{a}}_{1}=0.2$, $\bar{\tau }=0.15$. (**a**,**b**) are stationary states in in-phase mode with parameters ${\bar{a}}_{0}=0.2$, $v_{1}{}^{0}=0.1$, $v_{2}{}^{0}=0.5$. (**c**,**d**) are the vibration states in in-phase mode with parameters ${\bar{a}}_{0}=0.02$, $v_{1}{}^{0}=0.1$, $v_{2}{}^{0}=0.5$. (**e**,**f**) are stationary states in anti-phase mode with parameters ${\bar{a}}_{0}=0.2$, $v_{1}{}^{0}=0.1$, $v_{2}{}^{0}=-0.5$. (**g**,**h**) are the self-oscillation in anti-phase mode with ${\bar{a}}_{0}=0.02$, $v_{1}{}^{0}=0.1$, $v_{2}{}^{0}=-0.5$. There exist two synchronous modes of the system, namely, in-phase mode and anti-phase mode.

The time histories of mass displacements can be obtained by setting system parameters $\bar{K}$, $\bar{\alpha }$, $\bar{\beta }$, ${\bar{a}}_{0}$, ${\bar{a}}_{1}$, $\bar{\tau }$, $v_{1}{}^{0}$, $v_{2}{}^{0}$. The calculation results show that there are two synchronous modes in the system, namely in-phase mode and anti-phase mode, as shown in Figure 2. In calculation, the system parameter is set to: $\bar{K}=14$, $\bar{k}=8$, $\bar{\alpha }=-0.5$, $\bar{\beta }=0.1$, ${\bar{a}}_{1}=0.2$, $\bar{\tau }=0.15$. When ${\bar{a}}_{0}=0.2$, $v_{1}{}^{0}=0.1$, $v_{2}{}^{0}=0.5$, the two LCE fibers with the same initial velocity first vibrate in the same direction within the linear temperature field. Then, under the influence of damping, the amplitude of self-oscillation gradually decreases and finally stops on the upper side, as shown in Figure 2a,b. Although fibers convert heat into kinetic energy when heated, the converted kinetic energy does not keep them oscillating. When ${\bar{a}}_{0}=0.02$, $v_{1}{}^{0}=0.1$, $v_{2}{}^{0}=0.5$, the fiber will continue to vibrate in the temperature field, and finally evolve into self-oscillation, as shown in Figure 2c,d. In this case, the energy obtained from the temperature field is greater than the damping dissipation, so the self-oscillation is guaranteed. When ${\bar{a}}_{0}=0.2$, $v_{1}{}^{0}=0.1$, $v_{2}{}^{0}=-0.5$, the system can maintain the static mode of anti-phase mode, as shown in Figure 2e,f. As ${\bar{a}}_{0}=0.02$, $v_{1}{}^{0}=0.1$, $v_{2}{}^{0}=-0.5$, Figure 2g,h plot the displacement-time diagram and phase trajectory diagram in the anti-phase mode. A similar experimental phenomenon was reported by Ghislaine et al. [69], where two liquid crystal network oscillators interacted with each other driven by light and underwent synchronized in-phase and anti-phase oscillations in the steady state.

### 3.2. Self-Oscillation Mechanism

**Figure 3:**
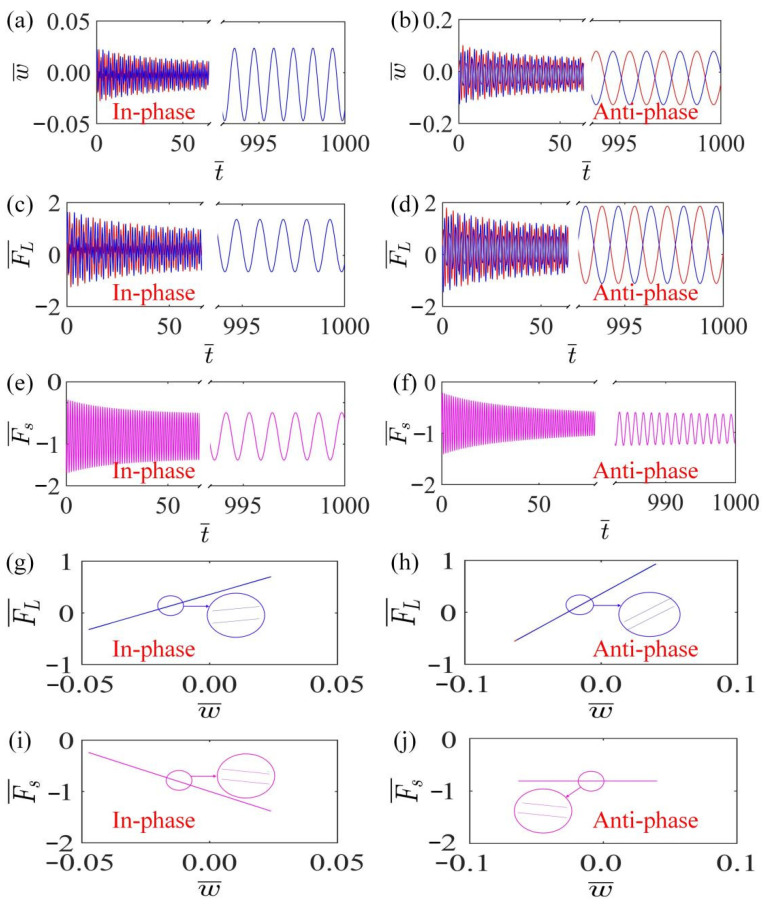
Self-oscillation mechanism of thermally responsive coupled LCE self-oscillators. The parameters for the in-phase mode are $\bar{K}=14$, $\bar{k}=8$, $\bar{\alpha }=-0.5$, $\bar{\beta }=0.1$, ${\bar{a}}_{0}=0.02$, ${\bar{a}}_{1}=0.2$, $\bar{\tau }=0.15$, $v_{1}{}^{0}=0.1$, $v_{2}{}^{0}=0.5$; the parameters for the anti-phase mode are $\bar{K}=14$, $\bar{k}=8$, $\bar{\alpha }=-0.5$, $\bar{\beta }=0.1$, ${\bar{a}}_{0}=0.02$, ${\bar{a}}_{1}=0.2$, $\bar{\tau }=0.15$, $v_{1}{}^{0}=0.1$, $v_{2}{}^{0}=-0.5$. (**a**,**b**) Time-history curves for in-phase and anti-phase modes; (**c**,**d**) change curve of driving force with time in in-phase and anti-phase modes; (**e**,**f**) spring force versus time curves for in-phase and anti-phase modes; (**g**,**h**) curves of the work done by the driving force for in-phase and anti-phase modes; (**i**,**j**) curves of the work carried out by the spring force for in-phase and anti-phase modes. The energy absorbed by the system from the external environment compensates for the damping dissipation, thus maintaining the self-oscillation of the system.

To further investigate the mechanism of self-oscillation of LCE fiber within the linear temperature field, Figure 3a,b plot the time-history curves of LCE fiber for in-phase and anti-phase modes, indicating that two LCE fibers oscillate periodically within the temperature field in in-phase and anti-phase modes. Figure 3c,e plot the curve of the tension of LCE fiber and spring changing with time in in-phase mode, indicating that the tension of LCE fiber and spring change periodically. Figure 3d,f plot the time-varying curves of the driving force and spring force in anti-phase mode, which indicate that the tension of LCE fiber and spring also maintain periodic changes in anti-phase mode.

Figure 3g,i show that in the in-phase mode, the LCE fiber and spring tension, along with the displacement, form hysteresis loops, and the region surrounded by the hysteresis loops represents the work done by the LCE fiber tension and spring force. The work done by the driving force of LCE fiber represents the energy input of the system, while the work done by the spring represents the work expended by the resistance. When the energy gain is equal to the resistance dissipation, the system will maintain self-oscillation. Figure 3h,j draw the hysteresis loops of the driving force of LCE fiber and spring force in anti-phase mode, which refers to the same energy compensation mechanism as the case of the in-phase mode.

### 3.3. Synchronization Mechanism

**Figure 4:**
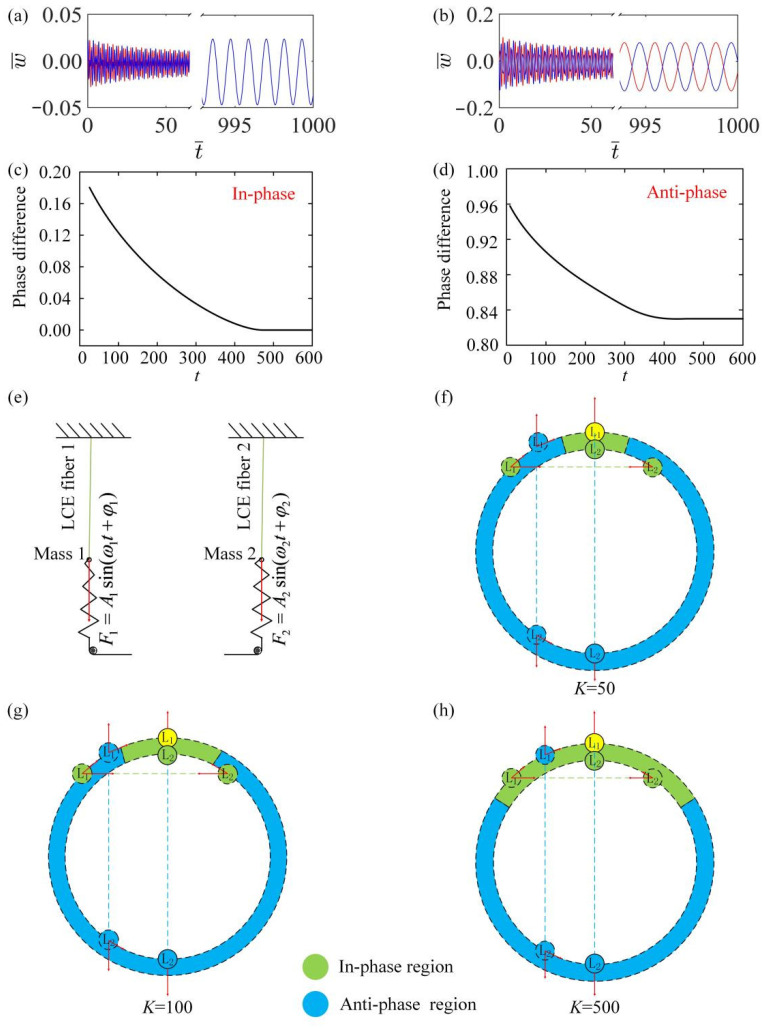
Synchronization mechanism of LCE spring oscillator. The parameters for the in-phase mode are $\bar{K}=14$, $\bar{k}=8$, $\bar{\alpha }=-0.5$, $\bar{\beta }=0.1$, ${\bar{a}}_{0}=0.02$, ${\bar{a}}_{1}=0.2$, $\bar{\tau }=0.15$, $v_{1}{}^{0}=0.1$, $v_{2}{}^{0}=0.5$; the parameters for the anti-phase mode are $\bar{K}=14$, $\bar{k}=8$, $\bar{\alpha }=-0.5$, $\bar{\beta }=0.1$, ${\bar{a}}_{0}=0.02$, ${\bar{a}}_{1}=0.2$, $\bar{\tau }=0.15$, $v_{1}{}^{0}=0.1$, $v_{2}{}^{0}=-0.5$. (**a**,**b**) Time history curve for in-phase and anti-phase modes; (**c**,**d**) phase difference curve for in-phase and anti-phase modes; (**e**) model of additional periodic forces applied; (**f**–**h**) schematic diagram of the interaction between phase attraction (in-phase region) and phase repulsion (anti-phase region) of two coupled harmonic oscillators. Two coupled harmonic oscillators always produce in-phase mode under strong interaction, while they may produce in-phase and anti-phase modes by changing the initial conditions under weak interaction.

To better study the mechanism of synchronization between two LCE fibers after self-oscillation in a linear temperature field, we plot some key physical quantities in the process of self-oscillation. Figure 4a,b draw the time-history curves for in-phase and anti-phase modes. Figure 4c,d, respectively, draw the change curve of the phase difference between fiber 1 and fiber 2 for in-phase and anti-phase modes. Figure 4c,d show that in the in-phase mode, the phase difference gradually decreases until it reaches zero, while in the anti-phase mode, the phase difference finally reaches a fixed value which is equal to half a cycle.

Through careful calculation, it is found that when the initial velocity directions of two self-oscillators are the same, the system will always develop into a synchronous mode. However, when the initial velocity direction is opposite, there is a critical LCE elastic coefficient that triggers a transition between in-phase and anti-phase modes. This result is similar to the existing experiment in that the elasticity coefficient can affect the synchronization mode of the system [69]. When the elastic coefficient of LCE is $\bar{K}<7100$, the system can be affected by the initial velocity, where the anti-phase synchronization mode occurs. When $\bar{K}\geq 7100$, the system always leads to an in-phase synchronous mode. In the case of weak interaction, i.e., $\bar{K}<7100$, the system can be likened to being acted on by external forces, as shown in Figure 4e. The system is divided into two LCE self-exciter separately for discussion. In the in-phase mode, each LCE oscillator is equivalent to applying an additional cycle force to another oscillator, which can be expressed as $F_{1}=A_{1}\sin(\omega_{1}t+\varphi_{1})$ and $F_{2}=A_{2}\sin(\omega_{2}t+\varphi_{2})$. When the periodic force F is consistent with the frequency of the harmonic oscillator, the synchronization phenomenon occurs. The same is true in the anti-phase mode.

Under the weak interaction, Figure 4f–h plots the changes in the system synchronization mode by changing the velocity of $L_{2}$ when the initial velocity $v_{1}{}^{0}=0.05$ of $L_{1}$ is fixed. As shown in Figure 4f–h, the ring represents the phase change in the process of movement. When the velocity of $L_{1}$ is unchanged, the position of $L_{1}$ does not change. When the velocity of $L_{2}$ is in the blue region, the system can achieve anti-phase mode, because the phases of the two repel each other. It can be seen from Figure 4f–h that an increase in the LCE elasticity coefficient leads to an increase in the synchronization region until, finally, the synchronization region covers all regions.

## 4. Parametric Analysis

In Equation (14), there are seven dimensionless parameters: $\bar{K}$, $\bar{k}$, $\bar{\alpha }$, $\bar{\beta }$, ${\bar{a}}_{0}$, ${\bar{\alpha }}_{1}$ and $\bar{\tau }$, which will affect the motion process of the system. This section discusses the influence of these system parameters on the amplitude and frequency of self-oscillation in in-phase and anti-phase modes.

### 4.1. Effect of LCE Elasticity Coefficient

**Figure 5:**
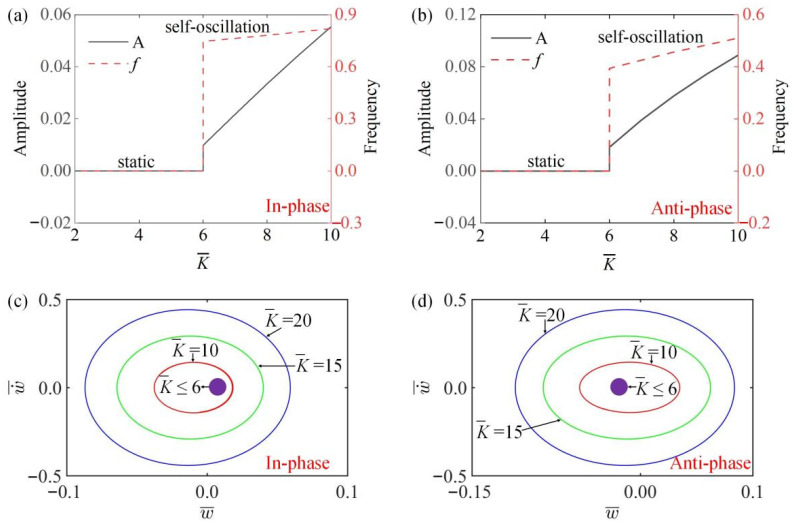
The influence of LCE elastic coefficient $\bar{K}$ on amplitude and frequency. The parameters for the in-phase mode are $\bar{k}=8$, $\bar{\alpha }=-0.5$, $\bar{\beta }=0.1$, ${\bar{a}}_{0}=0.02$, ${\bar{a}}_{1}=0.2$, $\bar{\tau }=0.15$, $v_{1}{}^{0}=0.1$, $v_{2}{}^{0}=0.5$; the parameters for the anti-phase mode are $\bar{k}=8$, $\bar{\alpha }=-0.5$, $\bar{\beta }=0.1$, ${\bar{a}}_{0}=0.02$, ${\bar{a}}_{1}=0.2$, $\bar{\tau }=0.15$, $v_{1}{}^{0}=0.1$, $v_{2}{}^{0}=-0.5$. (**a**,**b**) Time history curve of in-phase and anti-phase modes; (**c**,**d**) limit cycles of in-phase and anti-phase modes. The amplitude and frequency increase with the increase of $\bar{K}$.

In Figure 5a,b, the amplitude and frequency of the system change with the change of LCE fiber elasticity coefficient $\bar{K}$ in in-phase and anti-phase modes. Figure 5a,b show that when $\bar{K}\leq 6$, no matter in which mode, the system always achieves a static state, because the driving force ${\bar{F}}_{L}$ is less than the initial elastic force $F_{s}{}_{0}$ of the spring, and the LCE oscillator cannot vibrate, i.e., the amplitude and frequency are 0. When $\bar{K}>6$, the amplitude and frequency of the system gradually increase with the increase of $\bar{K}$. These results can be understood through the energy input and dissipation of damping. With the increase of $\bar{K}$, the driving force ${\bar{F}}_{L}$ gradually increases, and the energy supply from the linear temperature field gradually increases, so the amplitude and frequency of the self-oscillation increase. Figure 5c,d draw the limit cycles of different $\bar{K}$ in two modes. Figure 5c,d show that there is a limit value for the static state and self-excited state in the two synchronous modes, namely $\bar{K}=6$.

### 4.2. Effect of Spring Elasticity Coefficient

**Figure 6:**
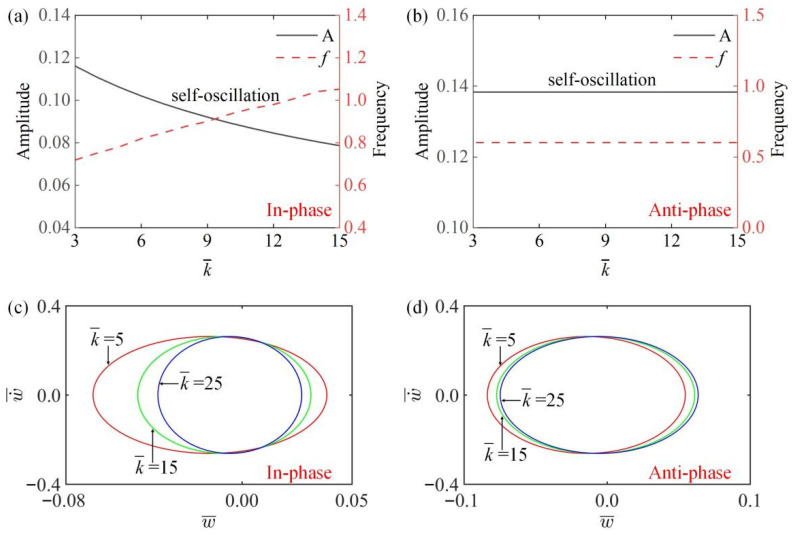
The influence of spring elastic coefficient $\bar{k}$ on amplitude and frequency. The parameters for the in-phase mode are $\bar{K}=14$, $\bar{\alpha }=-0.5$, $\bar{\beta }=0.1$, ${\bar{a}}_{0}=0.02$, ${\bar{a}}_{1}=0.2$, $\bar{\tau }=0.15$, $v_{1}{}^{0}=0.1$, $v_{2}{}^{0}=0.5$; the parameters for the anti-phase mode are $\bar{K}=14$, $\bar{\alpha }=-0.5$, $\bar{\beta }=0.1$, ${\bar{a}}_{0}=0.02$, ${\bar{a}}_{1}=0.2$, $\bar{\tau }=0.15$, $v_{1}{}^{0}=0.1$, $v_{2}{}^{0}=-0.5$. (**a**,**b**) Time history curve of in-phase and anti-phase modes; (**c**,**d**) limit cycles of in-phase and anti-phase modes. In the in-phase mode, the amplitude decreases and the frequency increases with the increase of spring elastic coefficient $\bar{k}$. In anti-phase mode, the amplitude and frequency are not affected by $\bar{k}$.

Figure 6a,b, respectively, draw the curves of the amplitude and frequency changing with different spring elastic coefficients $\bar{k}$ in the in-phase and the anti-phase modes. Figure 6a shows that in the in-phase mode, with the increase of spring elastic coefficient $\bar{k}$, the amplitude of the system gradually decreases and the frequency increases, because with the increase of $\bar{k}$, the damping dissipation increases, so the amplitude decreases gradually, while the increase of $\bar{k}$ makes the spring stiffness increase, so the frequency increases gradually. Figure 6b shows that the amplitude and frequency of the system remain basically unchanged in the anti-phase mode. This is because in inverting mode, the two LCE fibers move in opposite directions and at equal distances, so the total length of the spring connected to the lower end remains the original length, and the amplitude and frequency of the system remain the same. Figure 6c,d show the different limit cycles with different spring elastic coefficients $\bar{k}$ in two modes. The results show that the system is always in a vibration state in in-phase mode or anti-phase mode, and its motion mode is independent of $\bar{k}$.

### 4.3. Effect of Thermal Expansion Coefficient

**Figure 7:**
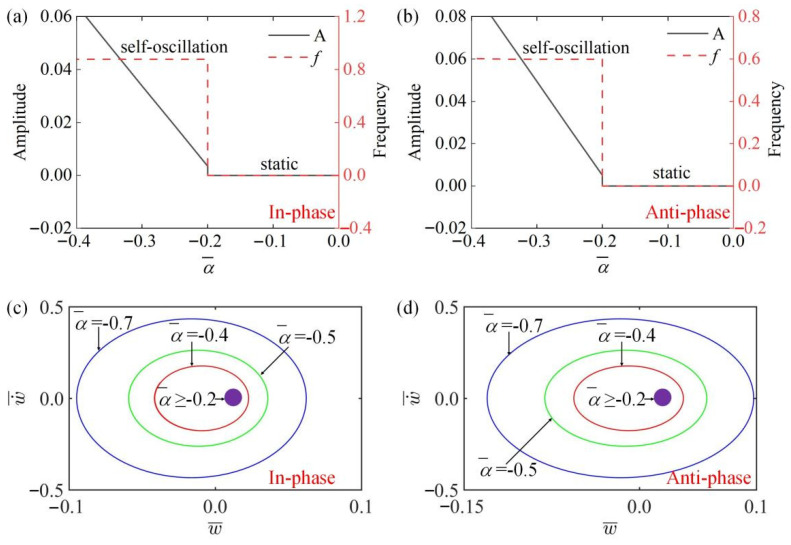
The influence of thermal expansion coefficient $\bar{\alpha }$ on amplitude and frequency. The parameters for the in-phase mode are $\bar{K}=14$, $\bar{k}=8$, $\bar{\beta }=0.1$, ${\bar{a}}_{0}=0.02$, ${\bar{a}}_{1}=0.2$, $\bar{\tau }=0.15$, $v_{1}{}^{0}=0.1$, $v_{2}{}^{0}=0.5$; the parameters for the anti-phase mode are $\bar{K}=14$, $\bar{k}=8$, $\bar{\beta }=0.1$, ${\bar{a}}_{0}=0.02$, ${\bar{a}}_{1}=0.2$, $\bar{\tau }=0.15$, $v_{1}{}^{0}=0.1$, $v_{2}{}^{0}=-0.5$. (**a**,**b**) Time history curve of in-phase and anti-phase modes; (**c**,**d**) limit cycles of in-phase and anti-phase modes. The amplitudes in two modes increase as the absolute value of $\bar{\alpha }$ increases, while the frequencies remain constant.

In Figure 7a,b, the amplitude and frequency of the system change with different thermal expansion coefficients $\bar{\alpha }$ in the in-phase and the anti-phase modes. It can be seen from Figure 7a,b that when $\left|\bar{\alpha }\right|\leq 0.2$, the system in two modes is in a static state, and the amplitude and frequency are zero. When $\left|\bar{\alpha }\right|>0.2$, the amplitude increases with the increase of the absolute value of $\left|\bar{\alpha }\right|$, and the frequency is unaffected, because the driving force gradually increases with the increase of $\left|\bar{\alpha }\right|$, so the amplitude gradually increases. Figure 7c,d draw the images of limit cycles changing with different thermal expansion coefficients $\bar{\alpha }$ in the in-phase and the anti-phase modes. Figure 7 shows that there is a critical value for both static state and vibration state in two modes, namely $\bar{\alpha }=-0.2$.

### 4.4. Effect of Temperature Gradient

**Figure 8:**
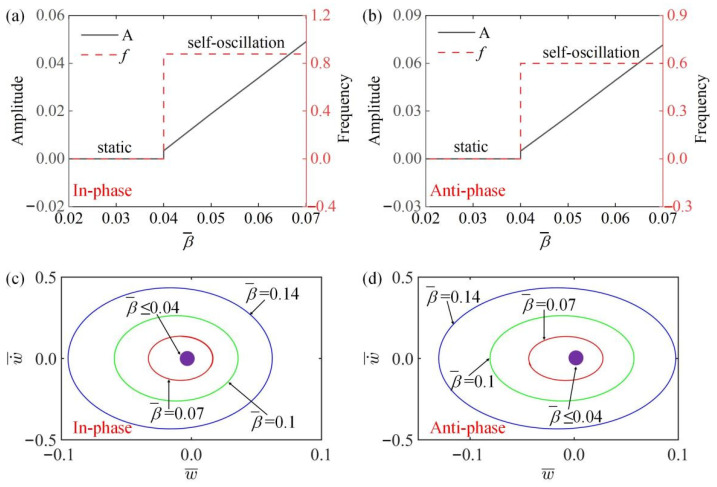
The influence of temperature gradient $\bar{\beta }$ on amplitude and frequency. The parameters for the in-phase mode are $\bar{K}=14$, $\bar{k}=8$, $\bar{\alpha }=-0.5$, ${\bar{a}}_{0}=0.02$, ${\bar{a}}_{1}=0.2$, $\bar{\tau }=0.15$, $v_{1}{}^{0}=0.1$, $v_{2}{}^{0}=0.5$; the parameters for the anti-phase mode are $\bar{K}=14$, $\bar{k}=8$, $\bar{\alpha }=-0.5$, ${\bar{a}}_{0}=0.02$, ${\bar{a}}_{1}=0.2$, $\bar{\tau }=0.15$, $v_{1}{}^{0}=0.1$, $v_{2}{}^{0}=-0.5$. (**a**,**b**) Time history curve of in-phase and anti-phase modes; (**c**,**d**) limit cycles of different modes. The amplitude of self-oscillation increases with the increase of $\bar{\beta }$, and the frequency remains unchanged.

Figure 8a,b plot the variation curves of amplitude and frequency of the system along with temperature gradient $\bar{\beta }$ in the in-phase and anti-phase modes. Figure 8a,b show that when $\bar{\beta }\leq 0.04$, the system is in a static state, where the amplitude and frequency are 0. On the contrary, when $\bar{\beta }>0.04$, the amplitude of two modes increases with the increase of $\bar{\beta }$, while the frequency is unchanged, which can be understood through energy input and damping dissipation because with the increase of $\bar{\beta }$, the temperature of the temperature field gradually increases, and the driving force ${\bar{F}}_{L}$ gradually increases, so the amplitude gradually increases. In Figure 8c,d, the limit cycles change with different temperature gradients $\bar{\beta }$ in two modes. It can be seen that there is a critical value $\bar{\beta }=0.04$ for both static state and self-oscillation state in in-phase and anti-phase modes.

### 4.5. Effect of the First Damping Coefficient

**Figure 9:**
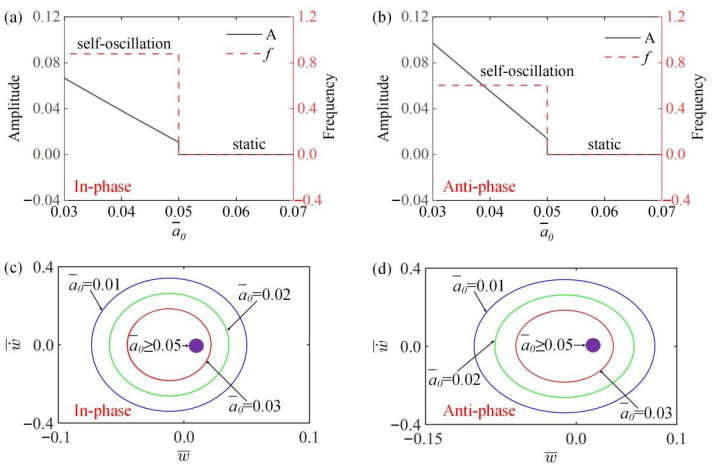
The influence of first damping coefficient ${\bar{a}}_{0}$ on amplitude and frequency. The parameters for the in-phase mode are $\bar{K}=14$, $\bar{k}=8$, $\bar{\alpha }=-0.5$, $\bar{\beta }=0.1$, ${\bar{a}}_{1}=0.2$, $\bar{\tau }=0.15$, $v_{1}{}^{0}=0.1$, $v_{2}{}^{0}=0.5$; the parameters for the anti-phase mode are $\bar{K}=14$, $\bar{k}=8$, $\bar{\alpha }=-0.5$, $\bar{\beta }=0.1$, ${\bar{a}}_{1}=0.2$, $\bar{\tau }=0.15$, $v_{1}{}^{0}=0.1$, $v_{2}{}^{0}=-0.5$. (**a**,**b**) Time history curve of in-phase and anti-phase modes; (**c**,**d**) limit cycles of two modes. The amplitude of self-oscillation decreases with the increase of ${\bar{a}}_{0}$, while the frequency remains unchanged.

Figure 9a,b show the variation curves of the amplitude and frequency with the change of the first damping coefficient ${\bar{a}}_{0}$ in the in-phase and anti-phase modes. It can be seen from Figure 9a,b that when ${\bar{a}}_{0}\geq 0.05$, the system is in static states, and the amplitude and frequency are 0. When ${\bar{a}}_{0}<0.05$, the amplitude of both the in-phase and the anti-phase modes decreases with the increase of ${\bar{a}}_{0}$, while the frequency is unaffected, because with the increase of ${\bar{a}}_{0}$, the work carried out by damping increases and the energy dissipation of the system increases, so the amplitude gradually decreases. In Figure 9c,d, the limit cycles change with the change of the first damping coefficient ${\bar{a}}_{0}$ in the two modes. The results show that the same limit value exists in the static state and vibration state of the in-phase and the anti-phase modes, namely, ${\bar{a}}_{0}=0.05$.

### 4.6. Effect of the Second Damping Coefficient

**Figure 10:**
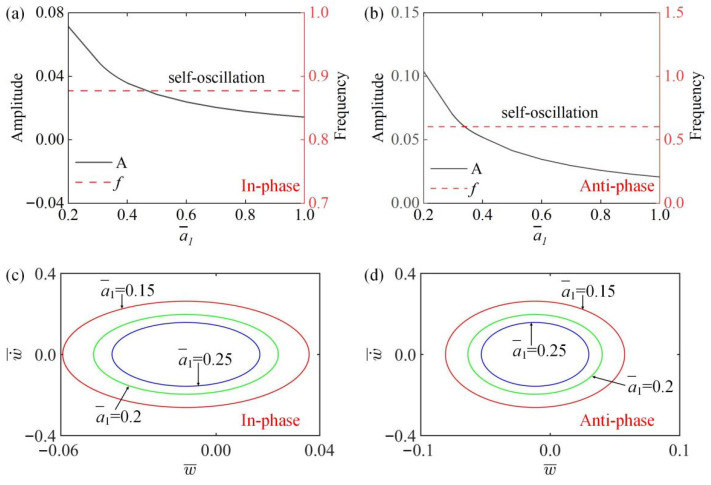
The influence of second damping coefficient ${\bar{a}}_{1}$ on amplitude and frequency. The parameters for the in-phase mode are $\bar{K}=14$, $\bar{k}=8$, $\bar{\alpha }=-0.5$, $\bar{\beta }=0.1$, ${\bar{a}}_{0}=0.02$, $\bar{\tau }=0.15$, $v_{1}{}^{0}=0.1$, $v_{2}{}^{0}=0.5$; the parameters for the anti-phase mode are $\bar{K}=14$, $\bar{k}=8$, $\bar{\alpha }=-0.5$, $\bar{\beta }=0.1$, ${\bar{a}}_{0}=0.02$, $\bar{\tau }=0.15$, $v_{1}{}^{0}=0.1$, $v_{2}{}^{0}=-0.5$. (**a**,**b**) Time history curve of in-phase and anti-phase modes; (**c**,**d**) limit cycles of in-phase mode and anti-phase mode. In both in-phase mode and anti-phase mode, the amplitude decreases as the second damping coefficient ${\bar{a}}_{1}$ increases, while the frequency remains constant.

Figure 10a,b show that the amplitude and frequency of the self-oscillation change with the change of the second damping coefficient ${\bar{a}}_{1}$ in the in-phase and the anti-phase modes. It can be seen from Figure 10 that the amplitude of the system decreases gradually as the second damping coefficient ${\bar{a}}_{1}$ increases in the in-phase and the anti-phase modes, while the frequency remains unchanged. This is because as ${\bar{a}}_{1}$ increases, the damping dissipation increases and so the amplitude gradually decreases. Figure 10c,d draw the images of the change of limit cycles with different second damping coefficients ${\bar{a}}_{1}$ in two modes. The results indicate that the system is always in a vibration state in in-phase and anti-phase modes, and its motion mode is independent of ${\bar{a}}_{1}$.

### 4.7. Effect of the Characteristic Time

**Figure 11:**
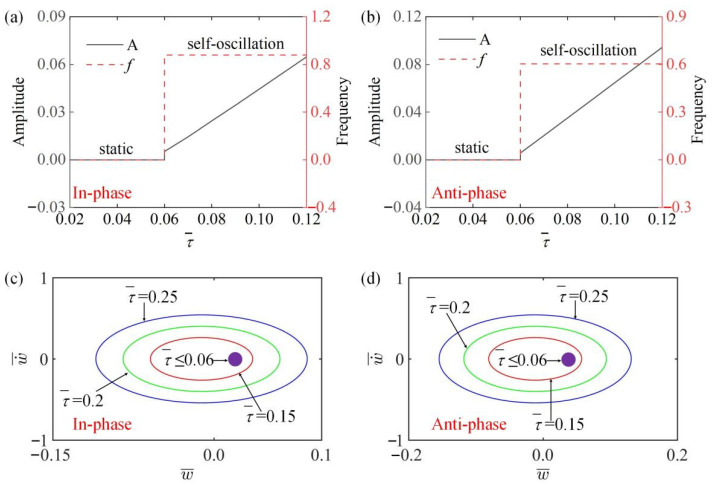
The influence of characteristic time $\bar{\tau }$ on amplitude and frequency. The parameters for the in-phase mode are $\bar{K}=14$, $\bar{k}=8$, $\bar{\alpha }=-0.5$, $\bar{\beta }=0.1$, ${\bar{a}}_{0}=0.02$, ${\bar{a}}_{1}=0.2$, $v_{1}{}^{0}=0.1$, $v_{2}{}^{0}=0.5$; the parameters for the anti-phase mode are $\bar{K}=14$, $\bar{k}=8$, $\bar{\alpha }=-0.5$, $\bar{\beta }=0.1$, ${\bar{a}}_{0}=0.02$, ${\bar{a}}_{1}=0.2$, $v_{1}{}^{0}=0.1$, $v_{2}{}^{0}=-0.5$. (**a**,**b**) Time history curve of in-phase and anti-phase modes; (**c**,**d**) limit cycles for different parameters. The amplitude of both modes increases with the increase of $\bar{\tau }$, while the frequency remains unchanged.

In Figure 11a,b, the amplitude and frequency change with different characteristic times $\bar{\tau }$ in the in-phase and the anti-phase modes. The results show that in both modes, when $\bar{\tau }\leq 0.06$, the system is in a static state and the amplitude and frequency are 0. At $\bar{\tau }>0.06$, the amplitude of the self-oscillation increases as $\bar{\tau }$ increases, and the frequency stays the same, because with the increase of $\bar{\tau }$, the heat transfer rate in LCE fiber increases, resulting in the gradual increase of driving force ${\bar{F}}_{L}$, so the amplitude gradually increases. Figure 11c,d draw the images of limit cycles changing with different temperature gradients $\bar{\tau }$ in in-phase and anti-phase modes. It can be obtained that there is a critical value $\bar{\tau }=0.06$ for triggering the self-oscillators in both modes.

## 5. Conclusions

The prevalence of synchronization and collective behaviors among self-excited coupled oscillators in nature necessitates investigation due to their inherent benefits, such as efficient energy harvesting, autonomous operation, and enhanced equipment portability. In this paper, based on thermally responsive LCE spring self-oscillators under a linear temperature field, the synchronous behavior of two coupled self-oscillators connected by springs is theoretically investigated. The mechanisms of self-oscillation and synchronization are theoretically investigated, integrating the well-established dynamic LCE model. According to the numerical findings, the system exhibits two synchronization modes: the in-phase mode and the anti-phase mode. Self-oscillations are sustained through a dynamic balance between damped dissipation and work carried out by the driving force.

The numerical findings indicate that the synchronization mode primarily depends on the interaction between two LCE self-oscillators. In cases of strong interaction with the elastic coefficient of LCE, the system consistently develops into the in-phase synchronous mode. However, when the interaction is weak, altering the initial conditions can lead to the in-phase and anti-phase modes. When the initial velocity direction of the two self-oscillators is the same or the initial velocity direction is opposite but the value is small, the system achieves the in-phase synchronous mode. On the contrary, as the initial velocity direction of the two self-oscillators is opposite and the relative value is large, the system evolves into an anti-phase synchronization mode.

In addition, the influences of the LCE elastic coefficient, spring elastic coefficient, thermal expansion coefficient and other system parameters on the synchronous mode, amplitude and frequency of the self-oscillations are systematically studied. The self-oscillation amplitude demonstrates a positive correlation with the increase of LCE elastic coefficient, thermal expansion coefficient, temperature gradient and characteristic time, while demonstrates a negative correlation with the increase of spring elastic coefficient and damping coefficient. Unlike existing work about self-oscillating synchronization systems based on active materials [7,70], this paper elucidates in detail the mechanism of the synchronization phenomenon. This study is expected to advance the comprehension of self-oscillator synchronization and provide its potential applications in diverse fields, including energy harvesting, power generation, detection, soft robotics, medical devices and micro/nanodevices. In addition, the research in this paper has the potential to be extended to large-scale synchronization systems containing a large number of coupled oscillators, which has a promising application in this field.

## Figures and Tables

**Table 1:** Material properties and geometric parameters.

Parameter	Definition	Value	Unit
L	original length of the LCE fiber	0.01–1	m
m	mass	0.01–0.1	kg
g	acceleration of gravity	9.8	m/s^2^
K	LCE elasticity coefficient	10–100	N/m
k	spring elasticity coefficient	10–100	N/m
α	thermal expansion coefficient	0.001–0.5	1/C
β	temperature gradient	20–1000	C/m
$a_{0}$	first damping coefficient	0.01–0.1	kg/s
$a_{1}$	second damping coefficient	0–0.2	kg/s
τ	characteristic time	0.001–0.1	s
$\rho_{c}$	heat capacity per unit length of LCE fiber	0.01–0.1	J/m^2^/C
h	heat transfer coefficient	1–20	W/m^2^/C

**Table 2:** Dimensionless parameters.

**Parameter**	$\bar{K}$	$\bar{k}$	$\bar{\alpha }$	$\bar{\beta }$	${\bar{a}}_{0}$	${\bar{a}}_{1}$	$\bar{\tau }$
**Value**	2–20	2–20	0–1	0.01–0.1	0.01–0.1	0.1–1	0.01–0.2

## Data Availability

Not applicable.
